# Prenatal care experiences among pregnant women with obesity in Wisconsin, United States: a qualitative quality improvement assessment

**DOI:** 10.1186/s12884-021-03629-4

**Published:** 2021-02-15

**Authors:** Danielle J. Hurst, Nicholas B. Schmuhl, Corrine I. Voils, Kathleen M. Antony

**Affiliations:** 1 University of Wisconsin School of Medicine and Public Health, 750 Highland Avenue, Madison, WI 53726 USA; 2 Department of Obstetrics and Gynecology, University of Wisconsin School of Medicine and Public Health, 1010 Mound Street, Madison, WI 53715 USA; 3 Department of Surgery, University of Wisconsin School of Medicine and Public Health K6/100 Clinical Science Center, 600 Highland Avenue, WI 53792 Madison, USA; 4 William S Middleton Memorial Veterans Hospital, 2500 Overlook Terrace, Madison, WI 53705 USA; 5 Division of Maternal-Fetal Medicine, Department of Obstetrics and Gynecology, University of Wisconsin School of Medicine and Public Health, 1010 Mound Street, 4th Floor, Madison, WI 53715 USA

**Keywords:** Obesity, Pregnancy, Weight-bias, Prenatal care, Provider weight bias

## Abstract

**Background:**

Stigma and bias experienced during prenatal care can affect quality of care and, ultimately, the health of pregnant women with obesity and their infants. We sought to 1) better understand the bias and stigma that women with BMIs ≥40 kg/m^2^ experience while receiving prenatal care, 2) gauge women’s interest in group prenatal education for women with obesity, and 3) gather feedback about their preferred weight-related terminology.

**Methods:**

We conducted and thematically content-analyzed 30 semi-structured interviews of women with BMIs ≥40 kg/m^2^ who received prenatal care at a university-affiliated teaching hospital in the Midwest region of the United States.

**Results:**

All women recalled positive experiences during their perinatal care during which they felt listened to and respected by providers. However, many also described a fear of weight-related bias or recalled weight-based discrimination. Women reacted favorably to a proposed group prenatal care option for pregnant women with obesity that focused on nutrition, physical activity, and weight management. Women rated “weight” and “BMI” as the most desirable terms for describing weight, while “large size” and “obesity” were rated least desirable.

**Conclusions:**

Many pregnant women with BMIs ≥40 kg/m^2^ experience bias in the prenatal care setting. Potential steps to mitigate bias towards weight include improving provider awareness of the experiences and perspectives of this population, expanding prenatal care options targeted towards women with high BMIs, including group care, and using patient-preferred weight-related terminology. Through the remainder of this manuscript, wherever possible, the term “high BMI” will be used in place of the term “obesity” to describe women with BMI ≥ 30 kg/m^2^ in order to respect the preferred terminology of the women we interviewed.

**Supplementary Information:**

The online version contains supplementary material available at 10.1186/s12884-021-03629-4.

## Background

According to birth certificate data, 27.8% of women in Wisconsin, United States who gave birth during 2011–2014 had a body mass index (BMI) ≥ 30 kg/m^2^ [1]. Published data on the prevalence of births among women with BMIs ≥40 kg/m^2^ in Wisconsin is lacking, but data from California show that the prevalence of births among women with BMIs ≥40 kg/m^2^ increased by 76% from 2007 to 2016 [2]. Women with BMIs ≥40 kg/m^2^ experience higher rates of adverse pregnancy outcomes such as gestational diabetes, hypertensive disorders of pregnancy, low cord blood pH, and post-cesarean complications compared to women with BMIs between 30 kg/m^2^ and 40 kg/m^2^ [3–6]. Women with high BMIs are also more likely to struggle with poor mental health, breast feeding, and post-partum weight management [7]. For these reasons, many pregnant women with high BMIs are considered high-risk and receive specialized care to improve maternal and fetal health outcomes [8].

Although obesity is prevalent in many populations, women with high BMIs often experience stigma in clinical settings, and the prevalence of experienced stigma is higher as BMI increases [9, 10]. Some women with high BMI feel they are given fewer choices regarding their care or feel that their care is grounded more firmly in weight-related bias than in their actual health needs [11]. During pregnancy, a time when most women gain weight, the impacts of stigma and bias are particularly complicated [12]. Depression resulting from stigma and bias are associated with delayed fetal growth, low birth weight, and premature births [12]. These concerns are compounded by the fact that healthcare providers often feel inadequately trained to discuss weight and obesity with pregnant women [13]. The implications of weight-related stigma have been studied in pregnant women and women with high BMIs, but there is a paucity of studies of pregnant women with high BMIs, specifically those with BMIs ≥40 kg/m^2^ [12, 14–16]. Existing studies show that stigma pertaining to weight has both short and long-term effects on patients in general [17]. The effects of these experiences include, but are not limited to, poor outcomes, long term stress exposures, and avoidance of clinical care [17].

Examining the experiences of patients, the language used by healthcare teams, and the terminology that is preferred by patients is essential to creating a healthcare environment that fosters inclusivity and patient comfort. A recent systematic review of 33 studies found that preferences for weight-related terminology differ across races, genders, and current weights [18]. In general, the terms “weight” and “unhealthy weight” were preferred over “obese” or “fat.” [18]

Another study found that the top source of interpersonal weight stigma was negative assumptions and ideas held by physicians about overweight individuals and people with high BMIs [19]. The second most prevalent form of stigma was inappropriate comments from physicians [19]. Although these studies investigated the perspectives of many patient populations with high BMIs who are diverse in age, gender, race and medical status, including pregnancy, [20–22], none have evaluated patient-preferred language among pregnant women with high BMIs.

Given the risks related to having a BMI ≥ 40 kg/m^2^ during pregnancy for both the mother and the developing fetus, additional screening or testing, such as growth ultrasounds, are often recommended [23, 24]. Indeed, prior investigations evaluating patient perceptions and knowledge of the risks of having a high BMI on pregnancy demonstrate consistent gaps in knowledge on the adverse effects of a woman having a high BMI on pregnancy outcomes [25]; knowledge gaps are also prevalent regarding gestational weight gain recommendations [21, 25–28]. Obstetric providers should inform pregnant women with BMIs ≥40 kg/m^2^ about the indications for any additional tests, the limitations of ultrasounds, and the risks of having a high BMI for maternal and fetal pregnancy outcomes [24]. Given the potentially harmful impacts of poorly executed provider-patient conversations about high BMIs during pregnancy, it is imperative to understand how healthcare professionals can best discuss high BMIs, weight complications, and weight gain with women. Whether this information is best conveyed one-on-one via physician-patient discussions or would be better discussed in a group setting or via a standardized video or other modality is unclear.

The purpose of this project was to identify ways to improve the quality of care for pregnant women with high BMIs receiving perinatal care. Through semi-structured qualitative interviews, we sought to 1) understand women’s perceptions of prior and recent clinical experiences where having a high BMI may have affected their care; 2) assess the need for prenatal educational opportunities for women with high BMIs; and 3) determine women’s preferences regarding weight-related language and terminology. While prior studies have evaluated perceived bias and knowledge gaps among pregnant women with high BMIs, ours uniquely queries pregnant women’s recommendations for educational and clinical interventions and preferences regarding weight-related language and terminology.

## Methods

This project was reviewed by the Institutional Review Board at Unity Point Health-Meriter Hospital and deemed to be exempt from full review, as the intent of the project was to inform and improve clinical practices and processes, and the findings were expected to directly affect institutional practice. All methods were performed in accordance with the institutional regulatory guidelines. Permission for this quality improvement initiative was obtained from the clinic manager and the medical director of the UnityPoint Health-Meriter Center for Perinatal Care (referred to here as “the clinic”) in Madison, Wisconsin, which is the clinical home of the Maternal-Fetal Medicine Division of the University of Wisconsin School of Medicine and Public Health. This hospital is located in an urban community but serves patient populations in rural surrounding areas throughout the state of Wisconsin. Patients interviewed reside in both rural and urban areas.

### Sample and recruitment

Investigators conducted a review of medical records to compile a list of women with 1) a pre-pregnancy body mass index of ≥40 kg/m^2^-who had delivered within the last 36 months; 2) attended at least one pre-natal visit at the clinic; and 3) completed their 6-week postpartum visit at the clinic. Exclusion criteria included 1) women who had delivered and were pregnant again due to a potential ongoing relationship with the clinic; 2) women whose medical charts noted the need for a medical interpreter due to lack of access to an interpreter; 3) women who experienced intrauterine fetal demise/stillbirth or neonatal death to ensure that recruitment was unlikely to trigger a significant stress response; and 4) women with developmental delay were also excluded. Interview prompts were created to achieve the aforementioned aims of improving care for pregnant women with obesity. The prompts related to preferred weight-related terminology were adapted from the literature [29–31]. All interview prompts were pilot tested on nurses at the clinic who were not otherwise involved in the project.

### Procedure

We contacted potential participants who met eligibility criteria by telephone between May 27th, 2020 and June 10th, 2020. Women who had given birth within the past 12 months were initially prioritized for recruitment. Because thematic saturation was not reached with this group, the pool was expanded to include those who had given birth within the past 36 months [32]. Women were called back at their preferred time and date if they indicated interest but were not available to talk at the time of the initial telephone contact.

### Interview format

After a woman agreed to participate, the interviewer began by introducing the purpose of the project and asking for verbal permission to audio-record the interview. The first section of the interview focused on women’s experiences receiving obstetric care. Questions queried participants about perceptions of respect and the listening skills shown by providers, discussions about weight and weight gain, women’s understanding of the effects of unhealthy weight on themselves and their babies during pregnancy, and ways in which the healthcare team could improve their communication about these topics. Interview prompts are listed in Table 1.

**Table 1:** Interview prompts regarding the experiences and perspectives of pregnant women with high BMIs

Topic	Question
**Women’s Experiences and Perspectives**	
General Experience	• Tell me about your experience receiving obstetrics care at [institution name]. • How much courtesy and respect do you feel that the providers, nurses, medical assistants, and front desk staff treated you with? • How much do you feel like the providers, nurses, medical assistants, front desk staff listened to you? • Tell me about any discussion you had with your provider about your weight. • How did your provider address your weight? • How did you feel when your healthcare provider first mentioned your weight? • Did you continue to feel that way or did your feelings change over the course of the discussion or your care? • Tell me about any discussion you had with your provider about gaining weight during pregnancy. • What did your provider say about gaining weight during pregnancy? • Did you provider explain that any of their recommendations were specifically related to your weight? For example, did they order extra ultrasounds, blood tests or lab tests, or fetal monitoring because of your weight?
Experiences and knowledge related to weight	Based upon your discussions and care with the healthcare team: • What is your understanding how your weight affects your health in general? • What is your understanding of how your weight affects pregnancy? • What is your understanding of how your weight affects the health of your baby?
Hospitalization, Labor, and Delivery	• Were you hospitalized on the antepartum unit prior to your delivery for reasons other than induction or management of labor? (If yes ask the following questions) • Tell me about your labor and delivery experience at Meriter. • Tell me about your postpartum experience at Meriter. • Are there any other experiences you’d like to share? • Tell me about any time that you felt that your weight affected your care or relationships with providers during pregnancy? • Tell me about anything your healthcare team could have done better to support you during your pregnancy? • Tell me about anything your healthcare team could have done better to help you manage weight gain during your pregnancy?

The second section asked women to rate the desirability of 10 weight-related terms using a 5-point response scale (1-very undesirable, 2-undesirable, 3-neutral, 4-desirable, and 5-very desirable). These terms, which were identified by prior studies reporting on patient preferences regarding weight-related terminology, included weight, heaviness, obesity, BMI, excess weight, excess fat, large size, unhealthy body weight, weight problem, and unhealthy BMI [29–31]. The term “fat” was excluded due to universal dislike of this term in prior studies and the potential to trigger perceptions of bias by utilizing this term. We also asked whether there was a most preferred term and used this weight-related term for the remainder of the interview. We interposed the third aim as the second section of the interview to allow us to gauge women’s preferred terminology after hearing about their clinical experiences; we also wanted to be able to use women’s preferred weight-related terminology for the remainder of the interview wherein we asked about potential future clinical models and improvements.

The third portion of the interview gauged interest in potential prenatal education programs aimed at helping women with high BMIs have a healthy pregnancy. Women were asked to provide feedback about potential clinical programming that may be offered to pregnant women with high BMIs in the future. Specifically, women were asked if they would consider participating in a group model of prenatal care and how they could be best supported in such a program. Women were asked about their interest in specific content for such a prenatal group and given the opportunity to suggest content-related ideas that were not proposed by the team (Table 2.) This section also inquired about women’s personal views about their weight, health, fitness, and satisfaction with their current weight. Additionally, women were asked why maintaining a healthy weight was important to them and how the importance of healthy weight relates to their health, their baby’s health, and their appearance.

**Table 2:** Interview prompts regarding future clinical models, classes, and education opportunities

Future Classes and Clinical Education Opportunities	
General Interest	• If group prenatal care were provided to women with ____(preferred term) during pregnancy, would you consider it? Why or why not?
Suggested Topics	• If there were such care models available, what you want to be included? • What about the following items … • Nutrition counseling at least once? • Nutrition at each visit with discussions of pitfalls and strategies that work for other group members? • Discussions about how to work physical activity into a busy daily routine? • Discussion about maintaining a healthy weight after your pregnancy or between pregnancies? • Actual physical activity as part of the visit or at the end? • Organized outings for physical activity like walks that allow strollers or other children or fundraiser event walks (if the entrance fee were waived)? • What do you think would be an appropriate title or name for such a group?

### Transcription and analysis

After the interviews were conducted, demographics such as ethnicity, race, comorbidities, and labor and delivery complications were extracted from delivery summaries in participants’ medical records. Race and ethnicity in the electronic health record are obtained via self-report. The interviewer transcribed the survey responses verbatim. All information, including interview audio, was stored in REDCap (Research Electronic Data Capture) [33].

We thematically analyzed transcripts using NVivo 12 Pro software (NVivo, QSR International Pty Ltd., 2020). Thematic saturation was reached, and five more interviews were conducted to ensure that women’s perspectives were well represented. Achievement of thematic saturation was determined by the interviewer and confirmed by the research team. Using NVivo, the data were organized into themes and sub-themes. Some themes pertained to topics of the interview prompts, which represented the interests of the team members, while other themes emerged from the participants’ statements [34]. The team member who performed the interviews transcribed the interviews, organized the data, and analyzed the content as described (DJH). Two additional members of the team reviewed the results of the content analysis (KMA and NS). Discrepancies and differences of opinion were resolved through discussion.

## Results

**Fig. 1:**
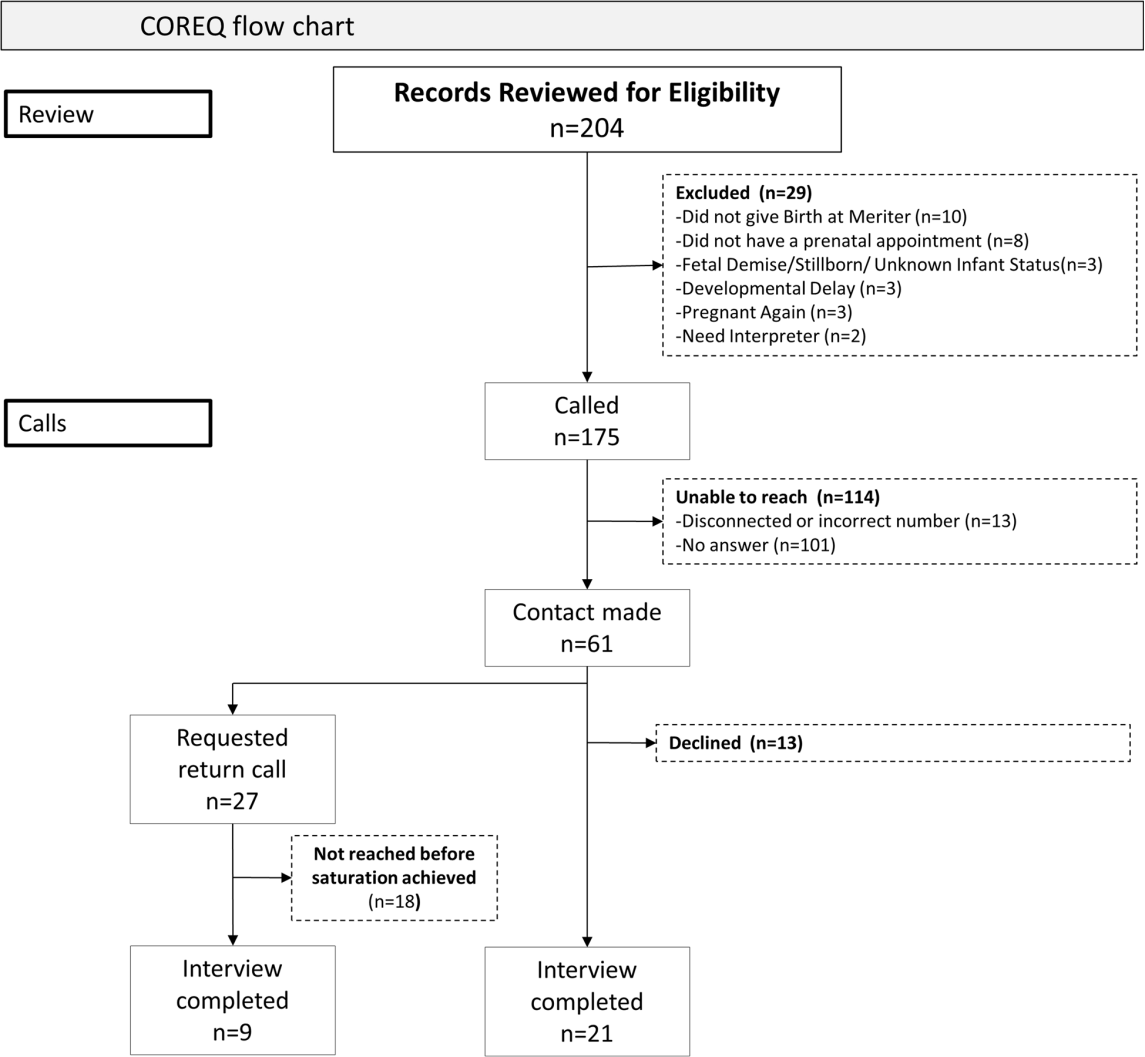
COREQ diagram. This is the flowchart in the COREQ (COnsolidated criteria for REporting Qualitative research) style for screening, enrollment, and interview completion

We reviewed 204 charts for eligibility. Of those, we determined 29 to be ineligible and were unable to reach 114 by telephone. We made telephone contact with 61 potential participants, 48 of whom consented to be interviewed. Of the 48, 18 consented and asked to be called back but were not reached again before saturation was achieved. A total of 30 participants were interviewed during either the first or second call. (Fig. 1.) Interview length ranged from 11 to 64 min, with a median interview time of 19.5 min.

The mean age of participants was 35.0 (SD 3.9), with the majority of participants (60%) aged between 35 and 40 years. Medical records identified participants as White (83.3%), African American (13.3%), or Asian (3.3%), with the majority being non-Hispanic (96.6%). The mean BMI of participants was 49.2 (SD 5.6). Forty percent of participants had a diagnosis of diabetes. Two-thirds (66.6%) of women had a hypertension diagnosis, and 40% of women had at least one mental health diagnosis during pregnancy. Additionally, 66.6% of participants had other health conditions noted in the admission or discharge diagnosis portions of their medical record. (Table 3.)

**Table 3:** Characteristics of the women interviewed (*N* = 30)

Demographics	***N*** (%)
**Age** Mean Age (Standard Deviation)	34.97 (SD 3.9)
20–24	1 (3.3%)
25–29	1 (3.3%)
30–34	9 (30%)
35–40	18 (60%)
40+	1 (3.3%)
**Race**	
White	25 (83.3%)
African American	4 (13.3%)
Asian	1 (3.3%)
**Ethnicity**	
Not Hispanic/Latinx	29 (96.7%)
Hispanic/Latinx	1 (3.3%)
**BMI** (kg/m^2^) Mean BMI (Standard Deviation)	49.2 (SD 5.55)
40–44.9	7 (23.3%)
45–49.9	11 (36.7%)
50–54.9	6 (20%)
55+	6 (20%)
**Diabetes**	
Any Type of Diabetes	12 (40%)
Type 2 Diabetes	7 (23.3%)
Pre-Diabetes	2 (6.7%)
Gestational Diabetes	5 (16.7%)
**Hypertension**	
Any Type of Hypertension	20 (66.7%)
Chronic Hypertension	6 (20%)
Gestational Hypertension	14 (46.7%)
Pre-Eclampsia without Severe Features	3 (10%)
Pre-Eclampsia with Severe Features	3 (10%)
**Mental Health**	
Any Mental Health Condition	12 (40%)
Depression	7 (23.3%)
Anxiety	7 (23.3%)
Other	7 (23.3%)
**Other Health Conditions**	
Women with an additional medical diagnosis	20 (66.7%)
Asthma	5 (16.7%)
Anemia	4 (13.3%)
Hypothyroidism	4 (13.3%)

The majority of women reported feeling neither satisfied nor dissatisfied with their current health and fitness and most felt at least somewhat dissatisfied with their current weight. Additionally, women reported that general health, pregnancy health, and the health of their baby were important reasons to maintain a healthy weight. Fewer people said that appearance was important.

In total, 20 themes and 72 subthemes were used to organize the data initially. Data were further categorized into three aims discussed below.

### Aim 1: understanding Women’s experiences and perspectives

Responses to the open-ended questions are represented by six themes, described below. Despite the preponderance of positive experiences, many women described at least one negative experience or an experience during their pregnancy which could be improved upon.

#### What women know

Several questions aimed to better understand participants’ knowledge and perceptions of how weight could affect their health, pregnancies, and babies. These responses offer insight into the ways in which providers talk about the potential effects of having a high BMI and how pregnant women understand this information.

All women reported some understanding of how their weight could affect their health. Some women discussed a need to lose more weight in order to be healthier, while others expressed the attitude that weight does not necessarily equate to health. Other women mentioned specific health conditions, such as high blood pressure or heart disease that could result from being overweight.

Most participants reported knowledge that weight could affect pregnancy. Gestational diabetes and blood pressure were two health concerns discussed specifically. Some women interpreted the question about weight and pregnancy as a reference to the effect of weight on fertility. These women understood that weight may affect a person’s chance of becoming pregnant. Additionally, most women reported an understanding that weight could affect their babies, noting birthweight and diabetes specifically. A few women did not remember having a discussion with their providers about how weight could affect their babies.

When asked about additional screenings or testing during prenatal care as a result of their high BMI, some recounted undergoing extra screenings but perceived that the screenings were in response to conditions other than having a high BMI.

*“All my extra stuff came from a Zika scare or mosquito scare … I'm also older, so I had extra for that. But they never said anything about weight … ”*

-Participant ID 1068

Others recalled extra screenings specifically related to having a high BMI.

*“Pretty much because I was obese, they wanted to monitor more frequently in case of my amniotic fluid or in case of gestational diabetes.”*

-Participant ID 1042

Some did not report any extra screenings.

Most participants reported having a discussion with their provider at the clinic or another provider about their weight, and most reported discussing weight gain during pregnancy. Women who discussed specific numbers most commonly recalled being told to gain 15 pounds, but other women said they were told to gain between 0 and 20 pounds. Some women reported losing weight during their pregnancies. A few women said they wished they had had more support regarding weight management during pregnancy. One woman described a conversation with her provider while expecting twins:

*“They gave me a lot of documentation around how much weight should you gain when you’re pregnant. They gave me a target range of how much they wanted me to gain. … They were telling me 10-22 (pounds).”*

-Participant ID 1187

#### Weight affects care

More than half of participants recalled a time when they felt their weight affected their care. These experiences ranged from poor communication on the part of a provider to pain during routine ultrasounds. Women reflected on experiences with the obstetrics team and other members of their healthcare teams. Some women perceived that they were weighed more frequently because of their weight, while others said they felt pressured into extra testing for conditions such as sleep apnea. One woman said she believed her weight and age contributed too strongly to the physician’s decision to order a sleep study:

*“I am overweight, that is true. However, all of the other criteria to apply to a sleep study, I didn’t have … But I was very much pressured to do a sleep apnea at-home sleep study because it was on the checklist of things that someone my age and weight should do rather than based on my actual answers to the screening questions.”*-Participant ID 1187

Some women described “scare tactics” used by their health care teams when discussing weight. One woman said her provider discussed alarming statistics that she did not feel pertained to her current health or pregnancy.

A few women said they felt that physicians lacked empathy or seemed “stand-offish.” One woman reflected on her experience during a follow-up appointment at the clinic and wondered if her experience would have differed if she were not overweight or obese:

*“At that follow up, I explained to her that I was really upset and felt dismissed and that I wasn't listened to … A consistent lack of understanding empathy and advocacy for patients, I'd say.”*

-Participant ID 1151

Some women recalled negative experiences with non-obstetric providers and ancillary staff while receiving perinatal care. Most of these women said they believed these experiences were directly related to their weight. Some women felt they were treated insensitively by ultrasound technicians or felt pain during ultrasounds, while others said their hospital stays were adversely affected by bias from workers ranging from food service professionals to resident physicians in non-obstetric fields such as anesthesia.

#### The patient as her own advocate

Some women said they did not always feel the care they received was adequate or appropriate for their needs. Women reported that they took some control over their care by initiating conversations about weight and requesting or denying various tests during and after pregnancy. Many women explained that in some situations they felt the need to advocate for their own care and did not feel heard. One woman described having to ask multiple times before receiving post-delivery pain medications. The same woman said she declined receiving certain tests and screenings that providers recommended when she felt the “only box she checked” was being overweight.

One woman described that, for her, talking about weight was not a delicate issue and that she often initiated the discussion.

*“It’s not a subject that I am particularly sensitive about. So, it might be easier to talk with me and often I bring it up because I know it’s an uncomfortable thing for people to bring up.”*

-Participant ID 1113

Some women also mentioned using online resources to supplement the care and advice they received. Women said they used the Internet to look up statistics or make decisions about their pregnancy, such as whether to pursue a vaginal delivery or cesarean delivery. Women reported using the Internet more often for weight-related pregnancy advice than for general pregnancy advice.

#### Missing the picture

Some women felt that their providers had not reviewed their entire charts or did not understand their pre-existing conditions well enough to provide adequate care. Some women wondered if diagnoses could have been made more efficiently if not for their weight. One woman felt that her weight was the primary focus of her healthcare and worried that providers might miss something if they only focused on weight:

*“So, my weight was part of the picture. It was the first thing that people saw and first thing people commented on, and half of the time it was the only thing people commented on.”*

-Participant ID 1153

#### Just say it

Some women wished that their providers had been more straight-forward when discussing weight and related care. Participants perceived a hesitance among some providers to discuss weight, or felt that their providers did not discuss connections between weight and other aspects of their health or care. Some women expressed that their providers could have been more transparent when approaching the subject of weight. Several explained that they expected their weight to inevitably affect their birth options, but that their providers did not disclose that information.

*“ … they tried three different ways to induce me and we still had to go with a c-section. It was towards the end where a nurse said, ‘Honey I can't believe you let them do all that to you.’ I just didn't know I could tell them no … So, I guess they could be a little more forthcoming.”*

-Participant ID 1204

Similarly, another woman said she underwent repeated cell-free DNA genetic screening, which continued to show inconclusive results. She explained that her healthcare team did not inform her that inconclusive test results could be related to her weight.

#### Prior experiences and fear

Some women shared experiences from past pregnancies that compared or contrasted with their experiences at the clinic. Some women said these prior experiences made them fearful of potential bias, affected the way they searched for future providers, or informed their decisions and discussions while receiving care at the clinic.

Some women recalled negative interactions with previous providers in which weight or weight-related care had not been discussed in a sensitive manner. One woman who felt pressured into further testing was told by her provider “… we are trying to avoid having a stillborn baby here …” after she expressed her frustration.

Women said they struggled to find providers who did not have a “fat bias” and wondered if any negative experiences during their care were due to provider bias against people who have high BMIs.

*“It’s unclear if the job is so hard and so there is so much information that you can never share it all with women or some women don’t want the information or if there is bias that because ‘you’re overweight you don’t take care of your health so we aren’t going to give you all the information is kind of the feeling you sometimes get.’”*

-Participant ID 1070

### Aim 2: future education opportunities

Women expressed a variety of opinions and suggestions regarding future patient educational opportunities and care models.

#### General interest

More than half of participants stated that a group prenatal care model would be helpful. Some who said it would be unhelpful said they worried that the location would be inconvenient for them, while others expressed concern about the time commitment. A few said it would be helpful during their first pregnancy but not for subsequent pregnancies. Others were simply uninterested.

#### Support for physical activity, nutrition, and weight management

Women were asked if they thought they would benefit from advice about incorporating physical activity into their busy schedules. Most women responded that this would be a helpful topic. Women said it would be helpful to include physical activity as part of the prenatal visit itself. Most women said it would be helpful for the program to include organized community outings to events such as fundraising walks.

Almost all participants said that it would be advantageous to discuss nutrition at least one time during group prenatal visits, while fewer stated that it would be helpful to discuss nutrition at each visit. Additionally, most women stated that it would be helpful to discuss weight management strategies to use during pregnancy and between pregnancies.

#### Other needs

A few women stated that discussions about breast feeding and its relationship to weight management or high BMIs would be helpful. Additionally, others stated that some form of accountability such as journaling or small quizzes would be helpful as part of such a group. A few women said they thought it would be helpful to address mental health and stress in the prenatal group by including activities such as meditation or discussions about mindfulness.

### Aim 3: weight related terminology

#### Preferred terminology

**Fig. 2:**
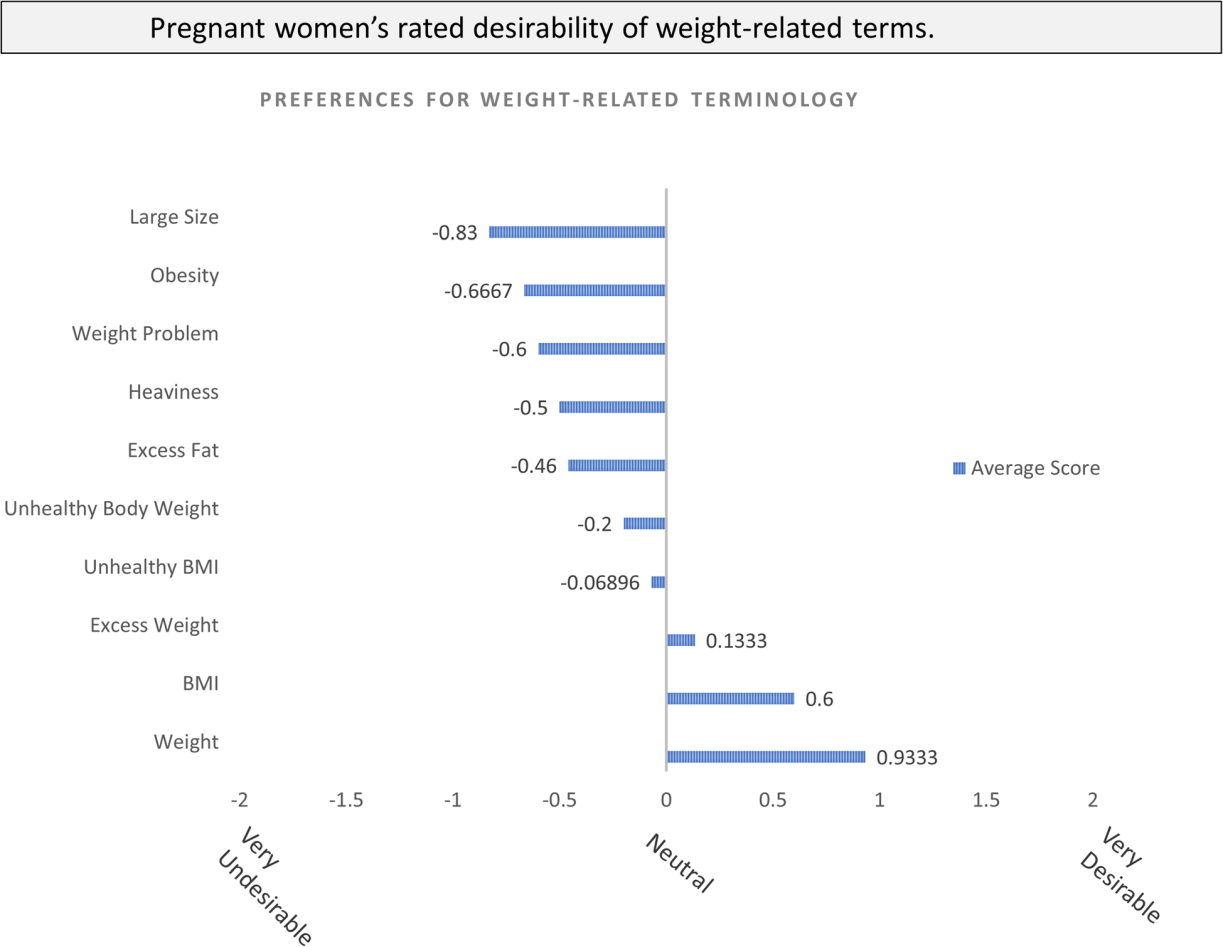
Pregnant women’s rated desirability of weight-related terms (scores range from (−2 (very undesirable) to + 2 (very desirable). BMI, body mass index

Among the 10 weight-related terms, the word “weight” was most frequently rated as “very desirable.” The word “obesity” was most frequently rated as “very undesirable.” (Fig. 2.)

Several women specifically mentioned disliking terminology used by their healthcare teams, including “obesity,” “BMI,” and “people your size.” A few women stated the opinion that BMI was not an appropriate concept to use to discuss weight.

*“I think the BMI that everyone is calculating is not appropriate to regular human beings... It's a little misleading. Someone might be at the same weight but have different measurements.”*

## Discussion

These findings reveal several impacts of weight and related stigma on the experiences of pregnant women with with BMIs ≥40 kg/m^2^ and provide insight regarding how the health care team can more effectively provide care to this population. Concepts identified here will help providers and other healthcare professionals to create an environment that is more accepting and understanding of pregnant women with high BMIs. Ultimately, we hope that this knowledge can be used to improve maternal and fetal health outcomes.

In summary, we found that women were at least somewhat knowledgeable about how weight can affect different aspects of pregnancy. In addition to affecting their pregnancies, many women said they felt that their weight may have affected the quality of their care. Women reported that they experienced bias and stigma from different members of their care teams, which took the forms of lack of empathy, lack of transparency, and scare tactics. Some women said they felt the need to initiate conversations or advocate for care that better fit their needs. This signifies an extra burden on pregnant women with high BMIs. Awareness of these women’s experiences and preferences allows us as medical providers to better understand the challenges facing pregnant women with high BMIs in the health care setting, and to develop solutions for eliminating bias and stigma in the clinic.

Women have reason to expect that they might experience bias and stigma before they even begin to seek prenatal care. Our participants reported difficulty finding physicians without “fat bias,” which is not surprising, given that a prior study found that 71% of patients with high BMIs have experienced at least one stigmatizing event in healthcare within the past year [35].

It is imperative that providers understand that the fears and worries of bias experienced by pregnant women with high BMIs in clinical settings can lead to detrimental health outcomes such as depression, avoidance of clinical care, and preterm birth [12, 16, 17]. Pregnant women with high BMIs have nearly a four times higher risk of pregnancy-related death compared to those who are not obese [36]. Additionally, women with high BMIs are more likely to delay pelvic exams and are three times more likely to report being denied care [37]. Providers caring for pregnant women with high BMIs should be aware of the high prevalence of patient-reported experiences with weight-related stigma and bias, and understand that the consequences have the potential to impact both maternal and fetal health.

Fears of bias and stigma due to weight often become realized experiences when a pregnant woman with a high BMI seeks care. Lack of empathy and understanding on the part of providers are not uncommon experiences in other patient populations with high BMIs [38]. This may be further complicated by the stereotype among some physicians that people with high BMIs lack understanding of, or responsibility for their own health and weight [39]. On the contrary, here we found that most women with high BMIs reported an understanding of the effect of their weight on their health, pregnancies, and babies.

Physician discomfort in talking about weight with women with high BMIs is likely a contributing factor to stigma [40]. Smith and colleagues found that while providers felt it was important to discuss the implications of having a high BMI with women, many were afraid to offend or embarrass their patients [40]. Healthcare providers in that study reported that they did not feel confident in giving advice to their patients with high BMIs [40]. In contrast, we found that most women could recall a conversation with their healthcare team about weight and weight gain during pregnancy, although the amount of recommended weight gain recalled did not always align with guidelines [41].

It is unknown if healthcare providers who often work with pregnant women with high BMIs feel more comfortable providing care than those who work with pregnant women with high BMIs less frequently. However, even healthcare providers with experience in providing care for people with high BMIs demonstrate high levels of bias [42]. Knowing that women prefer members of their healthcare teams to be more forthcoming in discussions about weight, and that some providers are uncomfortable providing the care that is necessary for pregnant women with high BMIs, clinics and hospitals should institute strategies to provide appropriate care that acknowledges the experiences, needs, and preferences of this patient population. These strategies should include education for all personnel who directly contact patients in any capacity.

Interest in group-based prenatal care or other patient educational models for pregnant women with high BMIs was high. Prior studies have also suggested that pregnancy is a time when women are willing to change their behaviors to be more health-promoting [43]. These suggestions and feedback will be considered by the clinic to improve the care of pregnant women with high BMIs.

The importance of using the preferred weight-related terminology cannot be underestimated. Studies have shown that using the wrong weight status terminology can offend women and weaken patient-provider relationships [44]. Professional medical organizations such the American Medical Association recommend using “non-stigmatizing” language when it comes to discussing high BMIs and weight with patients but do not make recommendations on specific language that would not be stigmatizing [45]. We found that most women dislike the term “obesity.” This aligns with seven other studies which aimed to understand what language is preferred among adults with high BMIs discussed in a recent systematic review [18]. Although these studies found that the term “weight” was rated as generally desirable and “obesity” was rated as generally undesirable, four of these studies found differences of opinion regarding other terminology we inquired about [18, 29, 30, 46]. This provides evidence that, for pregnant women with high BMIs, preferences for weight-related terminology may differ from the general patient population. Opportunities for women to indicate their preferred weight-related terminology on patient-intake forms, in addition to provider use of person-first language may be beneficial in creating a more inclusive clinical environment accounting for individual preferences [47].

In addition to understanding how providers can make their patients feel more comfortable in clinical environments, it is imperative to understand how hospital, city, and state policies can reduce bias and stigma experienced by people with high BMIs. Madison, WI is one of few cities in the country where laws that prohibit weight discrimination are in place to protect people with high BMIs in jobs, housing, and public resources [48]. A study conducted after the state of Michigan made weight-related employment discrimination illegal showed a specific reduction in perceived bias and stigma for women with with BMIs ≥35 kg/m^2^when over 1000 jobseekers were surveyed [49]. This evidence supports that systematic and institution-level changes positively impact the daily lives of people with high BMIs. In healthcare settings, changes such as introducing trainings that give health science students and providers an opportunity to learn and practice having conversations about weight, hiring standardized patients with more diverse BMIs, and focusing on patient centered communication are all potential solutions to reducing bias and stigma experienced in clinical studies [50]. The long-term effectiveness and impact of these strategies is yet to be well understood.

Our project has limitations. Our data collection and analysis were influenced by our backgrounds (medicine and psychology). The interviewer was a female medical student. While the student did not participate in the care of any participants, women may have been hesitant to admit certain things to someone they perceived to be a member of the medical team. An interviewer from outside of the medical field may have elicited different responses. Due to the characteristics of the population at this institution, we may have missed the experiences of other groups. For example, by focusing on women with with BMIs ≥40 kg/m^2^, we did not capture experiences of women with BMIs between 30 kg/m^2^ and up to 40 kg/m^2^, who may perceive stigma or terminology differently. Due to our exclusion criteria, we may have missed the experiences of women who speak languages other than English and those with developmental disabilities. Furthermore, we may have missed the experiences of women with non-White racial and Hispanic ethnic identities. Lastly, although we pooled from a large population of women, our project may be limited by our small sample size of interviews and potential discrepancies inherent in qualitative research.

## Conclusion

Health care teams can take steps to increase the inclusivity of clinic environments and care for pregnant women with high BMIs. This is imperative to reducing the bias and stigma experienced by this population. Ultimately, obstetric healthcare providers need to become more sensitive and confident when discussing the implications of high BMIs with women and acknowledge that women with high BMIs may have had negative experiences with healthcare providers in the past. In response to these experiences, women have found ways to initiate, advocate, and supplement their healthcare. In order to reduce the burden on the women receiving healthcare and to facilitate a more collaborative patient-provider relationship, obstetric providers should aim to understand and use the weight-related terminology preferred by pregnant women with high BMIs and incorporate the types of support this population needs and desires in their prenatal care. Future initiatives focused on eliminating weight-related bias and stigma and improving maternal-fetal outcomes should seek to understand the weight-bias held by obstetric providers, what kind of support providers need to reduce bias and increase transparency in discussions regarding high BMIs, and strategies to reduce weight-related bias in the prenatal setting.

## Supplementary Information

**Additional file 1.**

## Data Availability

Transcribed transcripts will be provided in a de-identified manner upon reasonable request to the corresponding author.

## Abbreviation

BMI: Body mass index
