# Supplementary material for: Prenatal care experiences among pregnant women with obesity in Wisconsin, United States: a qualitative quality improvement assessment
Source: BMC Pregnancy Childbirth. 2021 Feb 15;21:139. doi: 10.1186/s12884-021-03629-4 (PMC7885492; doi:10.1186/s12884-021-03629-4)
Supplement: Supplementary file 1 — Additional file 1. [file 12884_2021_3629_MOESM1_ESM.docx]

**Qualitative Obstetric/ Obesity QI Proposed Interview Questions for telephone interviews:**

[Before calling, please check obstetric discharge summary to ensure no fetal or neonatal death-we do not want to trigger significant distress in the interviewee.]

| Answered call (Y/N) |  |
| --- | --- |

**Script:**

Good [*morning/afternoon]*. I’m ____ and I am calling on behalf of the Meriter Center for Perinatal Care. We are wondering if you are willing to participate in a recorded telephone interview regarding your experience at our center. As part of this interview we will ask how we can improve our care and sensitivity for patients in the future. We specifically want to understand how healthcare providers discuss issues related to weight with pregnant patients. We know weight can be a sensitive topic so we want to learn how we can have positive, effective conversations with our patients. We would also like to ask about potential future programs which may improve women’s health during pregnancy and after. This interview is expected to last between 5 and 10 minutes. We are calling you because we see that you had at least one prenatal visit at the Meriter Center for Perinatal Care within the last 24 months.

Would you be willing to participate in a recorded interview answering such questions?

**For the rest of interview, I use the term provider when talking about your doctor, nurse practitioner, or physician’s assistant.**

| Willing to participate? (Y/N) |  |
| --- | --- |

If no,

🡪 ask “Can you briefly tell me why not” write down brief reason why.

If yes

🡪start recording and proceed to questions below.

[Questions for telephone interviewer: Record today’s date, recording start time, limited demographics, and recorder’s name]

| Today’s Date | __ __/__ __/ __ __ __ __  MM/DD/YYYY |
| --- | --- |
| Recording start time | __ __: __ __ [AM/PM] |

1. What is your first name?
2. What is your last name?
3. What is your date of birth?
4. What was the date of your most recent delivery or birth?

[Open ended questions:]

1. Tell me about your experience receiving obstetrics care at Meriter Center for Perinatal Care: [wait for answer]
   1. **How much courtesy and respect do you feel that the providers, nurses, medical assistants, and front desk staff treated you with?** [If no response or request for clarification, offer🡪] Did you feel that the providers, nurses, medical assistants, and front desk staff treated you with courtesy and respect?
      1. [Then]: Tell me more about that.
   2. **How much do you feel like the providers, nurses, medical assistants, and front desk staff listened to you?**
      1. [Then]: Can you **tell me more**?
2. We are calling to try to improve our care of obstetric patients whose weight is higher than recommended.
3. Tell me about any discussion you had with your provider about your weight.
4. (If yes) How did your provider address your weight?
5. (If yes) How did you feel when your health care provider first mentioned your weight?
   - - 1. Did you continue to feel that way or did your feelings change over the course of the discussion or your care?

(If necessary) Tell me more about that.

1. [Skip if the interviewee already talked about weight gain] Tell me about any discussion you had with your provider about gaining weight during pregnancy.
2. (If yes): What did your provider say about gaining weight during pregnancy?
3. Did you provider explain that any of their recommendations were specifically related to your weight? For example, did they order extra ultrasounds, blood tests or lab tests, or fetal monitoring because of your weight? [If necessary]: Tell me more about that.
4. Based upon your discussions and care with the healthcare team:
5. What is your understanding of how your weight affects your health in general?
6. What is your understanding of how your weight affects pregnancy?
7. What is your understanding of how your weight affects the health of your baby?
8. Were you hospitalized on the antepartum unit (or pregnancy floor) prior to your delivery for reasons other than induction or management of labor? [If yes, then ask the following questions]
   1. Tell me about your labor and delivery experience at Meriter.
   2. Tell me about your postpartum experience at Meriter.
   3. Are there any other experiences you’d like to share?
9. Tell me about any time that you felt that your weight affected your care or relationships with providers during pregnancy?
10. Tell me about anything your healthcare team could have done better to support you during your pregnancy?
11. [If they don’t mention weight gain] Tell me about anything your healthcare team could have done better to help you manage weight gain during your pregnancy?

We will next ask you a few questions about what words or terms you would prefer for your providers and others in the clinic or hospital to use if there is a need to refer to your weight.

Imagine you are visiting your **OB** provider. The nurse has measured your weight and the doctor will be in shortly to talk to you about weight gain recommendations for pregnancy and to provide other advice related to your weight and pregnancy. Doctors can use different terms to describe your weight. Please indicate how desirable or undesirable you would find each of the following terms if your doctor used it.”

The scale ranks from 5 being_ very desirable, 4 being _ desirable, 3 being_ neutral, 2 being_ undesirable, and 1 being_very undesirable)

1. “Good morning. I want to talk with you about your:”
   1. weight;
   2. heaviness;
   3. obesity;
   4. BMI;
   5. excess weight;
   6. excess fat;
   7. large size;
   8. unhealthy body weight;
   9. weight problem; and
   10. unhealthy BMI.^1,2^
   11. Are there any other terms not listed that you would prefer?
   12. Did any member of your healthcare team use terms for your weight that you did not like?
       1. (If yes) Do you mind sharing what those terms were so we can avoid them in the future?
   13. Of all the terms (or the terms you recommended), would you agree that ________ is your preferred term to use for healthcare visits and this interview? [OR if no clear preference, several the same score]: Which term would you prefer for healthcare visits and this interview?
   14. Anything else you would like to discuss about the terms listed above?
   15. Did the provider effectively explain why they care about [INSERT PREFERRED TERM HERE]__________?

We will next ask you a few questions about potential future classes or clinical education opportunities.

Group prenatal care is a new model of prenatal care where women with similar due dates gather together for two hour prenatal care visits. The first part of each visit includes a private visit with an **OB** provider who will measure the uterus, listen to the baby’s heart rate, and discuss your specific concerns with you. This portion would be the same as how you receive care now. After this part of the visit, each woman joins the group where general prenatal care topics are discussed, **like** what to expect at this point in pregnancy. Some prenatal care groups are geared toward women with specific medical conditions**, like** diabetes, so that women can get targeted dietary counseling and share recipes.

1. If group prenatal care were provided to women with (INSERT PREFERRED TERM HERE)________ during pregnancy, would you consider it?
   1. Why or why not?
   2. If there were such care models available, what would you want to be included, (even if you wouldn’t want such care for yourself)?
      1. First seek the interviewees ideas:
         1. ______
      2. What about the following items? [If they didn’t suggest the particular item]
         1. Nutrition counseling at least once
         2. Nutrition at each visit with discussions of pitfalls and strategies that work for other group members
         3. Discussions about how to work physical activity into a busy daily routine
         4. Discussions about maintaining a health weight after your pregnancy or between pregnancies?
         5. Actual physical activity as part of the visit or at the end
         6. Organized outings for physical activity **like** walks that allows strollers or other children or fundraiser event walk (if the entrance fee were waived)?
      3. What do you think would be an appropriate title or name for such a group?

🡪

We will finish by asking you a few questions about yourself.

1. How satisfied are you with your overall health and fitness?
   1. Very satisfied
   2. Somewhat satisfied
   3. Neither satisfied nor dissatisfied
   4. Somewhat dissatisfied
   5. Very dissatisfied
2. How satisfied are you with your current weight?
   1. Very satisfied
   2. Somewhat satisfied
   3. Neither satisfied nor dissatisfied
   4. Somewhat dissatisfied
   5. Very dissatisfied

To the extent that maintaining a healthy weight is important to you, what are the main reasons why it is important to you?

1. How important are the following reasons?
   - - - 1. Overall health?
         2. Healthy pregnancy?
         3. Healthy baby?
         4. Personal appearance?
2. Is there anything else you would like us to know?

Those are all the questions I have. Thank you for your time. Have a good day.

[After call, please complete the following limited demographics]

Limited demographics

| Interviewee first name |  |
| --- | --- |
| Interviewee last name |  |
| Interviewee Self-reported Race (from Epic) |  |
| Interviewee education level (from Epic) |  |
| Medical co-morbidities (YES/NO to each) |  |
| -Type 1 diabetes |  |
| -Type 2 diabetes |  |
| -Prediabetes |  |
| -Gestational diabetes |  |
| -Chronic hypertension |  |
| -Gestational hypertension |  |
| -Preeclampsia |  |
| -Other (specify) |  |
| Delivery information |  |
| -Mode of delivery (vaginal or Cesarean) |  |
| -Gestational age at delivery |  |
| -Delivery complications (specify) |  |
